# Prevalence of functional constipation among Palestinian preschool children and the relation to stressful life events

**DOI:** 10.1371/journal.pone.0208571

**Published:** 2018-12-06

**Authors:** Denise Froon-Torenstra, Elise Beket, Ali M. Khader, Majed Hababeh, Arwa Nasir, Akihiro Seita, Marc A. Benninga, Maartje M. van den Berg

**Affiliations:** 1 Emma Children’s Hospital/Academic Medical Center, Amsterdam, the Netherlands; 2 United Nations Relief and Works Agency for Palestinian refugees in the Near East, Headquarters Amman, Jordan; 3 University of Nebraska Medical Center, Omaha, NE, United States of America; 4 Haaglanden Medical Center, The Hague, the Netherlands; London School of Economics and Political Science, UNITED KINGDOM

## Abstract

**Aim:**

Increasing evidence exists with respect to the relation between stressful life events and functional constipation (FC). We aimed to investigate the prevalence of FC in Palestinian refugee preschool children and to determine if stress and trauma exposure are risk factors of FC in these children.

**Methods:**

From November 2013 until May 2014, a cross-sectional survey was conducted in West Bank, Gaza and Jordan. Mothers of 862 Palestinian refugee children aged 7–48 months were interviewed on defecation pattern, socio-economic factors and the child’s exposure to traumatic events.

**Results:**

Twelve percent of the Palestinian refugee children fulfilled the criteria for FC. The prevalence of constipation was significantly lower in Gaza compared to Jordan (2% vs. 17%, p <0,001). Living in Gaza was associated with lower odds of FC (OR 0,08, 95% CI 0,03–0,20). Trauma exposure was associated with higher odds of FC (OR 1,19, 95% CI 1,06–1,35), however only a small number of children had been exposed to traumatic events.

**Conclusion:**

The overall prevalence of FC in Palestinian preschool children is comparable to prevalence rates among older children worldwide. In this age group stressful life events and trauma exposure seem not to play an important role in the development of FC.

## Introduction

Functional constipation (FC) is a common functional gastro-intestinal disorder (FGID) among children, with a median worldwide prevalence of 9,5% (95% CI 7,5–12,1), ranging from 0,5% to 32,5% [1–2]. Multiple factors play a role in the etiology of pediatric FC, and they vary among different age groups [3–4]. The main cause for the development of constipation in toddlers is retentive posturing, resulting in thickening of stool, and is often related to stubbornness or anxiety for (painful) defecation [5–6].

A recent systematic review showed that children with FC are significantly more exposed to stressful life events than healthy children [7]. Additionally, abuse and traumatic experiences can contribute to the development of FGIDs in older children and adults [8]. Whether stress plays a role in the development of FC in younger children is unknown.

In the aftermath of the 1948 Arab-Israeli war, Palestinian refugees in Jordan have built a reasonably stable life in contrast to the occupied Palestinian territory (West Bank and the Gaza Strip), where children are more subjected to the conflict. Consequently, Palestinian refugee children living in the occupied Palestinian territory are at greater risk of stressful life events [9].

The aim of this study is to compare the prevalence of FC between the Palestinian refugee preschool children in Jordan, West Bank and Gaza. We hypothesize that a stressful environment and trauma exposure are associated with higher prevalence rate of FC among preschool children living in the occupied Palestinian territories.

## Patients and methods

A cross-sectional survey was conducted in 12 health centers of United Nations Relief and Works Agency for Palestinian Refugees in the Near East (UNRWA) located in Jordan, West Bank and Gaza. These UNRWA health centers provide primary child health care to Palestinian refugees until the age of five years, and they reach an immunization coverage of 95%-99% [10]. Health centers included in the study were: Amman New Camp, Zarqa, Baqa’a, Irbid, Jabaliah, Gaza Town, Khan Younis, Rafah, Balata, Tulkarem, Hebron and Aamary. Healthy children between 7 and 48 months of age who were visiting the well-baby clinic with their mother were eligible for enrolment. Older children were not included in the study due to the low visiting rate in health centers.

Based on previous research, we used the expected prevalence rate of 10% for functional constipation [11–12]. With a confidence level of 95%, a power of 80% and a precision to the nearest 2%, the estimated sample size was 862 children. Half of the sample was from Jordan and the other half from the occupied Palestinian territory. The sample was stratified for age according to the following four age-categories: 7–12 months; 13–24 months; 25–36 months and 37–48 months. For each age category, we divided the sample size among the health centers in proportion to the total number of registered children at each health center.

### Data collection

Data were collected by face-to-face interviews in Arabic. One nurse in Jordan and four nurses in West Bank, and a medical doctor in Gaza administered the interviews. Due to travel restrictions it was impossible to use one interviewer for all locations. All interviewers received a three-hour training including background information, consent procedure, methods of interviewing and data collection. Due to the high prevalence of illiteracy in this population, verbal consent was obtained of the mothers. Refusal of consent was documented on the data collection sheet.

The questionnaire consisted of 2 parts; part 1 was administered by direct interview of the mother. This part included questions about demographic data, medical history of the child, the child’s defecation pattern and domestic violence. Part 2 was self-administrated and consisted of questions about mother’s and father’s demographics, social-economic situation and the exposure to traumatic events of the child during his/her life.

The first part of the questionnaire was based on the validated questionnaire for child and adolescent functional constipation (Questionnaire on Pediatric Gastrointestinal Symptoms—Rome III Version QPGS- RIII) [13]. FC was defined by ROME III criteria for toddlers [4]. Stool retention was defined as stool withholding, large diameter stools were defined as stools that clogged the toilet, and fecal incontinence was only documented for toilet trained children. In general, toilet systems in the three different regions were comparable. Rectal mass was documented when there was a history of rectal mass discovered during rectal exam by a clinical doctor in the past. No physical examination was conducted at time of interview. Bristol stool scale was used to define hard bowel movements [14].

The child’s exposure to traumatic life events was documented and scored by using the Gaza Traumatic Checklist [15–16]. This checklist consisted of a questionnaire filled in by the mother, mothers who were illiterate received help from the interviewer. The split half reliability of the scale was *r* = .73. The internal consistency of the scale, calculated using Cronbach’s alpha, was α = .72 [16]. The score was analyzed as a total score and as a categorical variable, ranging from low exposure (<5), moderate exposure (5–9) to high exposure (>9) [15].

The standardized questionnaire was first written in English and afterwards translated in Arabic by a medical doctor and a communication expert, both were native Arabic speakers and had good English skills. The questionnaire was pilot tested on two representative samples and was adjusted based on the results from the pilot testing. For English and Arabic copy of the questionnaire see S1 and S2 Appendices.

### Statistical analysis

Data was entered and analyzed using SPSS 21.0. Descriptive statistics were used to assess prevalence rates and 95% confidence intervals of FC in the whole sample and per location. Chi-Square equations and Mann Whitney U test were used to determine the differences in demographic, social-economic data and traumatic events between locations.

Multiple logistic regression was used to determine potential risk factors for the outcome FC. Dependent variable was FC, the main independent variable was location category (Jordan, West Bank and Gaza). Univariate logistic regression was first performed to assess relevant independent variables. Potential confounding variables (demographics, social-economic variables, variables related to psychosocial stress and trauma) were all included in the final model. P-values less than 0,05 were considered statistically significant.

### Ethical consideration

This study was conducted under jurisdiction of UNRWA, following the ethics regulations of the United Nations. The study and consent procedure has been approved by the local ethical committee of UNRWA Headquarter Ethics Office in Jordan. In addition, ethical approval was obtained from the Nebraska Medical Center Institutional Review Board.

## Results

From November 2013 until May 2014 a total of 862 mothers of Palestinian refugee children were interviewed; 430 from Jordan, 217 from West Bank and 215 from Gaza. The median age of the children was 24,8 months (IQR 12,6–38,8), of whom 49,9% (95% CI 46,8–53,1) were male. There was no difference in age and gender between the three locations.

**Fig 1:**
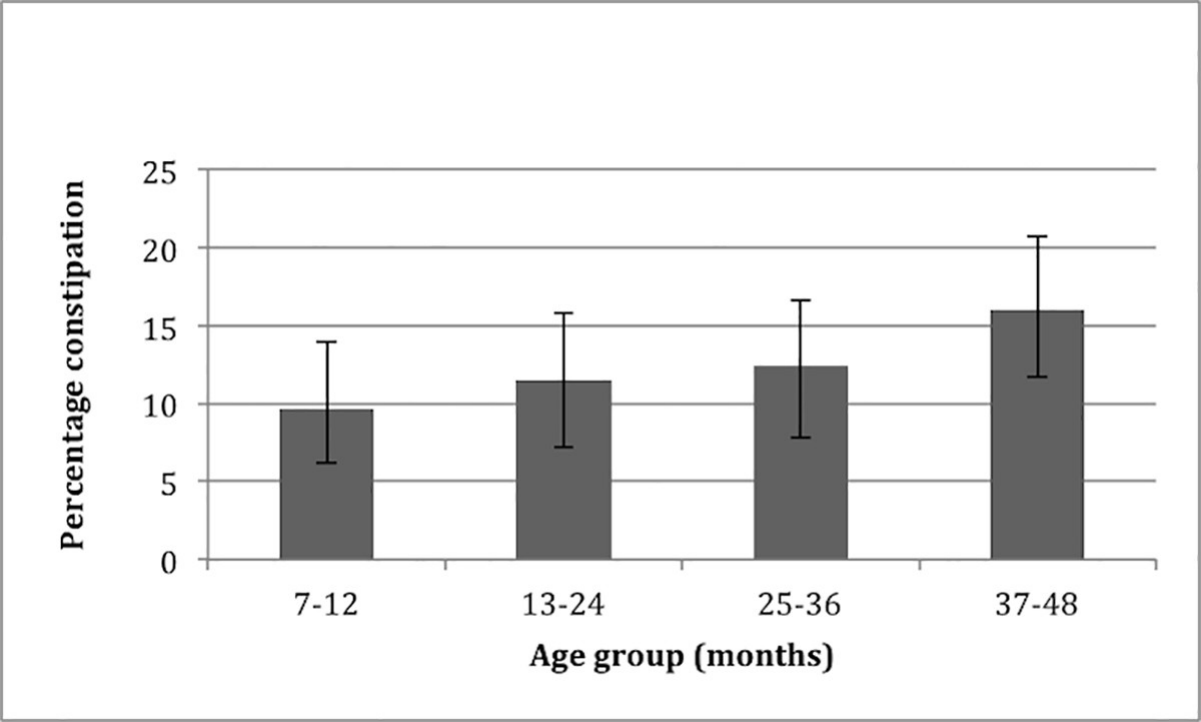
Prevalence of functional constipation per age group. Error bars depict 95% confidence interval.

Table 1 depicts the prevalence of FC and the occurrence of each ROME III criteria per location. The prevalence of constipation was significantly lower in Gaza compared to Jordan and the West Bank (2% vs. 17% and 13%, respectively, p <0.001). No significant difference was found in prevalence of constipation between the different age groups (Fig 1).

**Table 1:** Prevalence of functional constipation, and defecation pattern in total sample and per location in percentage (95% confidence interval).

	All	Jordan	West Bank	Gaza
Functional constipation	12,4 (10,5–14,7)	16,5 (13–20,2)	13,4 (8,8–18,2)	2,3 (0,5–4,7)*
Defecation frequency <3/week	2,1 (1,2–3,1)	2,6 (1,2–4,2)	2,4 (0,5–4,8)	0,9 (0,0–2,3)
Fecal incontinence ≥1/week	1,8 (0,9–2,7)	2,6 (1,2–4,2)	0,5 (0,0–1,4)*	1,4 (0,0–3,3)
Stool withholding behavior	5,5 (4,0–7,2)	7,5 (5,1–10,3)	5,3 (2,4–8,2)	1,9 (0,5–3,8)*
Painful bowel movement %(95% CI)	20,0 (17,3–22,7)	23,5 (19,7–27,5)	16,8 (12,0–22,6)**	16,0 (11,1–21,1)**
Rectal mass at physical exam	2,7 (1,8–3,8)	0,9 (0,2–1,9)	7,2 (3,8–11,1)*	1,9 (0,5–3,8)
Large diameter stools	20,6 (17,9–23,30)	25,4 (21,4–29,6)	28,8 (22,6–35,1)	2,8 (0,9–5,2)*
Toilet trained for bowel movement	44,1 (40,7–47,6)	45,2 (40,7–50,1)	41,3 (34,1–49,0)	44,6 (37,1–51,6)
Mushy/watery stools	26,5 (23,5–29,5)	32,8 (28,4–37,5)	18,0 (13,1–23,7)**	22,3 (16,9–28,5)*

*: Significant different compared to Jordan, P< 0,001.

**: Significant different compared to Jordan, P< 0,05.

Table 2 depicts the demographic, social-economic variables, stress related variables and traumatic exposure among the three locations. Mothers and their children from the West Bank and Gaza experienced more domestic violence compared to mothers and children living in Jordan. Significantly more mothers in Gaza were forced to move to another house because of security reasons compared to Jordan. Additionally, the median trauma score was significantly higher in Gaza compared to Jordan. The proportion of moderate and high trauma scores was also higher in Gaza compared to Jordan.

**Table 2:** Social-economic data and variables related to psychosocial stress and trauma in the different locations.

	Variable		Jordan	West Bank	Gaza
Social variables	Mothers age—median (IQR)		28 (24–32)	27 (23–31)	27 (23–31)
	Mothers marital age—median (IQR)		19,5 (18–22)	19 (18–21)	19 (17–22)
	Mothers education—years—median (IQR)		12 (9–12)	12 (10–14)*	12 (10–14)**
	Fathers age—median (IQR)		33 (29–39)	32 (28–37)	31 (27–36)*
	Fathers martital age-median (IQR)		26 (23–29)	25 (23–28)	24 (22–27)*
	Fathers education-median (IQR)		10 (8–12)	10 (8–12)	12 (9–14)**
Economic variables	Mother unemployed % (95% CI)		95,5 (93,4–97,4)	90,2 (86,0–93,9)*	95,8 (92,8–98,1)
	Father unemployed % (95% CI)		4,7 (2,8–6,8)	6,2 (3,3–9,5)	30 (23,9–35,7)**
	Income low < 300 JD or < 1500 shekel % (95% CI)		61,6 (57,0–65,9)	34,9 (28,8–41,2)**	67 (60,9–73,2)
	Income doesn't meet essential needs % (95% CI)		68,1 (63,5–72,4)	66,2 (60,2–72,7)	73 (66,5–79,1)
	Family has loans % (95% CI)		52,6 (48,4–57,5)	64,8 (58,3–71,3)*	72,4 (66,2–78,2)**
Housing variables	Place of residence % (95% CI)	Urban area	54,3 (49,9–59,1)	48,8 (42,4–55,8)	54,4 (47,9–61,6)
		Rural area	15,9 (12,4–19,2)	16,1 (11,8–21,2)	6,0 (3,3–9,3)
		Refugee area	29,7 (25,134,0)	35,0 (28,4 41,0)	39,5 (31,9–46,5)*
	Forced to move % (95% CI)		7,7 (5,3–10,2)	6,9 (3,7–10,6)	34,9 (28,4–41,4)**
Stress-related	Violence against mother % (95% CI)		19,4 (15,7–23,2)	34,6 (28,6–41,5)**	40 (33,5–46,0)**
	Violence against child % (95% CI)		2,6 (1,3–4,2)	41 (34,4–47,7)**	41,9 (35,3–48,4)**
	Bad/very bad relationship husband % (95% CI)		3,0 (1,4–4,7)	2,8 (0,9–5,1)	4,2 (1,9–7,0)
	Bad/very bad relationship family members % (95% CI)		4,0 (2,3–6,1)	1,4 (0,0–3,2)	7,4 (4,2–11,2)**
Traumatic events	Traumascore total—median (IQR)		0 (0–1)	0 (0–1)*	2 (0–4)**
	Traumascore % (95% CI)	Low	98,6 (97,4–99,5)	96,8 (94,0–98,6)	80,5 (74,9–86,0)**
		Moderate	1,4 (0,5–2,6)	3,2 (1,4–6,0)	16,3 (11,6–21,4)**
		High	0	0	3,3 (0,9–5,6)**

*: Significant different compared to Jordan. P<0,05.

**: Significant different compared to Jordan. P<0,001.

Multiple regression analysis showed that living in Gaza is related to lower odds for having FC (p<0,001, OR 0,05, 95% CI 0,02–0,18). Higher odds for FC was seen in families who had an income that did not meet the essential needs (p = 0,002, OR 2,45, 95% CI 1,39–4,31). Other demographic-, social- or economic-, and stress related factors were not significantly predictive or protective for FC. (Table 3)

**Table 3:** Multivariate logistic regression.

Independent variable		p-value	OR (95% CI)
Location	Jordan	*reference*	
	West Bank	0,329	0,76 (0,42–1,34)
	Gaza	<0,001	0,05 (0,02–0,18)
Age group	7–12 months	*reference*	
	1–2 years	0,410	1,3 (0,68–2,62)
	2–3 years	0,570	1,2 (0,61–2,46)
	3–4 years	0,071	1,99 (0,94–4,22)
Male		0,266	1,23 (0,83–2,01)
Number of siblings		0,987	0,99 (0,67–1,48)
Birth order		0,389	1,21 (0,78–1,87)
Mothers age		0,159	0,90 (0,79–1,04)
Mothers marital age		0,205	1,10 (0,95–1,27)
Mothers education in years		0,504	0,97 (0,88–1,06)
Mother unemployed		0,430	1,66 (0,47–5,93)
Fathers age		0,871	1,01 (0,89–1,13)
Fathers marital age		0,731	1,02 (0,90–1,15)
Fathers education in years		0,079	1,07 (0,99–1,16)
Income doesn't meet essential needs		0,002	2,45 (1,39–4,31)
Traumascore		0,018	1,18 (1,03–1,35)
Violence experienced by mother		0,381	1,07 (0,92–1,23)
Violence experienced by child		0,997	1,00 (0,87–1,14)
Bad/very bad relationshsip with husband		0,707	0.78 (0,21–2,83)
Bad/very bad relationship with other family members		0,039	0,60 (0,36–0,97)
Place of residence	Urban area	*reference*	
	Rural area	0,259	0,67 (0,33–1,34)
	Refugee camp	0,227	0,73 (0,44–1,22)

Hosmer & Lemeshow Chi-square 9,52 p-value 0,30

The median trauma score in children with FC was 1,0 (IQR 0,0–2,0) and was not significantly different from children without FC where the median trauma score was 0,0 (IQR 0,0–2,0). Multiple logistic regression analysis showed that more trauma exposure was related to FC (p = 0,018 OR 1,18, 95% CI 1,03–1,35). (Table 3) However, only a small number of children reported high trauma scores (7 children, all living in Gaza), but none of these children reported FC. Moderate trauma scores were reported by 47 children, of whom 10,4% (95% CI 2,1–18,8%) had FC.

## Discussion

This cross-sectional survey investigated the prevalence of functional constipation (FC) among preschool children in the Middle East and the relation to stressful life events with FC. The overall prevalence of FC in infants and toddlers of Palestinian refugees was 12%, which is comparable to the median prevalence of 9,5% reported worldwide [1–2]. Stressful life events and trauma exposure seem not to play an important role in the development of FC in this age group.

To date, only a few studies have investigated the prevalence of FC in infants and toddlers. A retrospective chart review found a prevalence of 2,9% in the first years of life, and a prevalence of 10,1% in the second year of life [17]. Comparable data were identified by a recent cross-sectional survey of 1447 mothers throughout the US, showing a prevalence of FC of 4,7% among infants and 9,4% among toddlers [12]. A cross-sectional study from Jordan showed that compared to older children, infants and toddlers visited the Pediatric Gastroenterology clinic more often for constipation [18]. Our study showed an increasing prevalence of FC with increasing age. However, no significant difference was found between age groups.

By studying a refugee population with the same culture and traditions, living in different environments with various levels of stress and exposure to traumatic events, we aimed to evaluate if traumatic and stressful events were potential risk factors for FC. In contrast to our hypothesis, children in Gaza showed a significantly lower chance of developing FC. The prevalence of 2.2% among preschool children in Gaza is among the lowest estimates worldwide [11]. Differences in environmental factors and diet may explain the low prevalence found in our survey. People in Gaza may have less access to healthy food and clean water [9], which in theory could lead to a higher prevalence of diarrhea. However, mushy or watery stools were less frequently reported by mothers in Gaza compared to Jordan. No information about dietary habits was collected during the survey. Multiple logistic regression analysis did not reveal any variables with a protective role in the development of FC. The use of multiple personnel performing the interviews could have biased our results. However, all personnel received similar training and there were no big differences found in results between different interviewers. Another limitation is that the QPGS-RIII questionnaire was not validated for this specific age group and population. Furthermore, Bellini et al. assessed the subjective perception of being constipated with the presence of ROME III criteria by performing a survey among patients referred to a gastroenterologist due to chronic constipation and the people accompanying them. Among the subjects who did not consider themselves constipated, 27,3% of patients and 14,4% of the non-patients were considered constipated according to the ROME III criteria [19]. This underlines the limitation of criteria based diagnosis of constipation.

A recent systematic review reported that stressful life events, ranging from separation from a friend to living in a war-effected area, occurred more frequently among children with constipation compared to healthy children between 3 to 18 years old [7]. In Sri Lanka a significant higher prevalence of FC was found among children between 10 and 16 years of age who were living in a war-effected area [20–21]. With our survey in younger children we were not able to confirm these observations. Although multiple logistic regression showed that increasing trauma score could be a risk factor for functional constipation, we question the relevance of this finding. Stress related variables and trauma exposure were indeed more prevalent in Gaza compared to Jordan. However, only a small number of children were categorized with high and moderate trauma scores and they did not report constipation more frequently.

The finding that stressful life events and trauma exposure seem not to play an important role in the development of FC in children below 4 years of age, may be explained by the different underlying mechanisms of FC in younger and older children. Transition from breast milk to infant formula, the introduction of solid food [1] and retentive posturing during toilet training play an important role in development of FC in young children [5–6]. Possibly, older children are more aware of stressful and traumatic experiences and therefore are more at risk to the negative health consequences caused by stress. It is also suggested that functional gastrointestinal disorders in older children and adults can be triggered by a stressful experience, mainly in individuals with a predisposing genetic background and who have been primed by a sensitizing event that occurred early in life [22]. Mechanisms underlying this theory include changes in the enteric nervous system, immune system, or microbiota that occur over time [23].

In conclusion, the overall prevalence of FC in Palestinian refugee children between 7 months and 4 years of age was 12%. Trauma exposure and stressful events seem not to play an important role in the development of FC in the preschool age. Long-term follow-up of our cohort is needed to determine if these children, are more susceptible to FC later in life. Moreover, more research is needed to determine the factors that contribute to the development of FC in this particular age group.

## Supporting information

S1 Appendix Questionnaire in English. (PDF)

S2 Appendix Questionnaire in Arabic. (PDF)
