# Supplementary material for: Prevalence of functional constipation among Palestinian preschool children and the relation to stressful life events
Source: PLoS One. 2018 Dec 6;13(12):e0208571. doi: 10.1371/journal.pone.0208571 (PMC6283531; doi:10.1371/journal.pone.0208571)
Supplement: S1 Appendix — (PDF) [file pone.0208571.s001.pdf]

Case number:……………………….

# Assessment of constipation among Palestine refugee children aged 7 months to 4 years

Date:…………………………………….......  Child Health Record No ..............................
UNRWA Health Center..........................................................................................................
Location: ___ Jordan, ____ West Bank, ____ Gaza
Age categories: ___ 7-12 mo, ___ 13-24 mo, ___25-36 mo, ___ 37-48 mo

Part 1 and 2 of the questionnaire will be completed by interview of the mother.
Part 3 and 4 of the questionnaire are self-administered and will be completed by the mother.

## PART 1

**Information about the child:**

1. Name :.........................................................................................
2. Date of Birth :.........................................................................................
3. Sex : ___ male, ___ female
4. How many siblings : number of females…………number of males.............
5. What is the birth order? :...........................................................
6. Current weight :……………………………  grams
7. Current height :……………………………… cm
8. a. Growthcurve (see Child Health Record)? ____ normal, ___ abnormal
   b. If abnormal, which growthcurve (see Child Health Record)
   1. ___ overweight (> 2SD weight for length)
   2. ___ underweight (< 2SD weight for age)
   3. ___ stunting (< 2 SD length for age)
   4. ___ wasted (< 2SD weight for length)

9. General medical history :……………………………………………………………………………
10. Is your child mentally developing normal: __ no, __ yes

## PART 2

This section asks about your child's bowel movements. There are many words for bowel movements, such as "poop," "stool," "BMs," and "going to the bathroom for number 2." Your family may use another special word when they talk about poops.

11. In the last 2 months, how often did your child usually have poop?
    1. __ 2 times a week or less → continue with question 12
    2. __ 3 to 6 times a week
    3. __ Once a day
    4. __ 2 to 3 times a day
    5. __ More than 3 times a day

    (options 2–5: continue with question 13)

12. How long has your child had bowel movements 2 times a week or less?
    1. __ Since less than 2 month
    2. __ Since 2 to 6 months
    3. __ Since 6 months to one year
    4. __ Since 1 - 2 years
    5. __ Since 2 - 3 years
    6. __ Since 3 - 4 years

13. In the last 2 months, what was your child's poop usually like?
    1. __ Type 1: Very hard (separate hard lumps)
    2. __ Type 2: Hard
    3. __ Type 3: Dry and lumpy
    4. __ Type 4: Smooth and soft
    5. __ Type 5: Very soft blobs
    6. __ Type 6: Mushy
    7. __ Type 7: Watery
    8. __ It depends (his/her poops are not always the same)

14. In the last 2 months, did your child have pain during defecation?
    1. __ Never
    2. __ Once in two months
    3. __ 1 to 3 times per month
    4. __ Once a week
    5. __ More than once a week
    6. __ Every day

15. In the last 2 months, did your child have to strain during bowel movements?
    1. __ Never
    2. __ Once in two months
    3. __ 1 to 3 times per month
    4. __ Once a week
    5. __ More than once a week

6. __ Every day

16. In the last 2 months, did your child have a poop that was so big that it clogged the toilet (large diameter stool)?
    1. __ No
    2. __ Yes

17. Some children hold in their poop even when there is a toilet available. They may do this by stiffening their bodies or crossing their legs.
    In the last 2 months, when at home, how often did your child try to hold in a poop?
    1. __ Never
    2. __ Once in two months
    3. __ 1 to 3 times per month
    4. __ Once a week
    5. __ More than once a week
    6. __ Every day

18. a. Is your child toilet trained (clean) at home and outside home?
    1. __ No → continue with question 21
    2. __ No, only at home → continue with question 21
    3. __ Yes

    b. If yes, at what age did your child became clean? ……………………………months

19. For children who became completely toilet trained (clean).
    In the last 2 months, how often was your child's underwear stained or soiled with poop?
    1. __ Never
    2. __ Once in two months
    3. __ 1 to 3 times per month
    4. __ Once a week
    5. __ More than once a week
    6. __ Every day

20. For children who became completely toilet trained (clean).
    In the last 2 months, how often was your child's underwear soiled with urine in daytime?
    1. __ Never
    2. __ Once in two months
    3. __ 1 to 3 times per month
    4. __ Once a week
    5. __ More than once a week
    6. __ Every day

21. In the last 2 months, how often did your child pass blood with stools?
    1. __ Never
    2. __ Once in two months
    3. __ 1 to 3 times per month
    4. __ Once a week
    5. __ More than once a week
    6. __ Every day

Did your child have the following symptoms during the last 2 months?

| | | |
|---|---|---|
| 22. Abdominal pain | 1. __ no | 2. __ yes |
| 23. Vomiting | 1. __ no | 2. __ yes |
| 24. Loss of appetite | 1. __ no | 2. __ yes |
| 25. Loss of weight | 1. __ no | 2. __ yes |

26. In your opinion, is the stool pattern a problem for your child?
    1. ___ No
    2. ___Yes

27. Did a doctor or nurse ever examine your child and say that your child had a huge poop inside?
    1. __ No
    2. __ Yes

28. a. Did you ever visit an UNRWA health center because your child had stool problems?
    1. __ Yes
    2. __ No, we went to another clinic or hospital
    3. __ No

b. If yes, at what age?..................................months.

c. If yes, what tests did the doctor/nurse do (more answers are possible)?
    1. __ Examination abdomen
    2. __ Anal and rectal exam
    3. __ Blood test :…………………………………
    4. __ X-ray :…………………………………
    5. __ ultrasound :…………………………………
    6. __ other :…………………………………
    7. __ no test

29. a. Has your child ever been treated for constipation by an UNRWA doctor/nurse?
    1. __ Yes
    2. __ No
    3. __ No, self treatment or pharmacy advise

b. The treatment included (more answers are possible):

1. __ dietary advice:
    a. __ eating more fibres
    b. __ eating more fruits
    c. __ drinking more water
    d. ___ other………………………………………
2. __ child behavior advice:
    a. __ toilet training,
    b. __ punishing
    c. __ rewarding
    d. __ other…………………………..
3. __ oral laxatives:
    a. ___ tablets form ……………………………………..
    b. ___ syrup form ………………………………………
    c. ___ powder form ……………………………………
4. __ rectal laxatives:
    a. ___ glycerin supp
    b. ___ other supp
    c. ___ rectal enema
5. __ herbal remedies, specify…………………………………
6. __ other…………………………………………………………

30. In the last year, were you subject to physical or verbal violence in your own house?

1. __ Never
2. __ Once a year
3. __ More than once a year
4. __ Once a month
5. __ More than once a month
6. __ Once a week
7. __ More than once a week
8. __ Daily

31. In the last last, was your child subject to physical or verbal violence in your own house?

1. __ Never
2. __ Once a year
3. __ More than once a year
4. __ Once a month
5. __ More than once a month
6. __ Once a week
7. __ More than once a week
8. __ Daily

## PART 3

### Information about the family

32. Mother's age in years :....................................................................
33. Mother's marital age in years :…………………………………………………………………..
34. Mother's years of education :....................................................................
35. Mother's employment:
    1. Unemployed
    2. Unskilled worker
    3. Skilled (hand)worker
    4. Civil employee
    5. Merchant
    6. Professional with a degree
    7. Other occupation…………………………………………

36. Father's age in years :....................................................................
37. Father's marital age in years :…………………………………………………………………..
38. Father's years of education :....................................................................
39. Father's employment:
    1. Unemployed
    2. Unskilled worker
    3. Skilled (hand)worker
    4. Civil employee
    5. Merchant
    6. Professional with a degree
    7. Other occupation……………………………….…………

40. Household income:

Jordan
1. ___ less than 300 JD
2. ___ 300-500 JD
3. ___ more than 500 JD

West Bank / Gaza
1. ___ less than 1500 shikel
2. ___ 1500 – 2500 shikel
3. ___ more than 2500 shikel

41. Does your income meet your essential needs?
    1. ___ no
    2. ___ yes

42. Does your family have any loans?
    1. ___ no
    2. ___ yes

43. How is your relationship with your husband?
    1. ___ Very good
    2. ___ Good
    3. ___ Bad
    4. ___ Very bad

44. How is your relationship with other family members (parents, parents-in-law, brothers and sisters) who are living in your house?
    1. ___ Very good
    2. ___ Good
    3. ___ Bad
    4. ___ Very bad
    5. ___ No other family members live in my house

45. Place of residence:
    1. ___ refugee camp
    2. ___ urban area
    3. ___ rural area

46. a. Was your family forced to change their place of residence because of security reasons or other related reasons?
    1. ___ no
    2. ___ yes

    b. If yes, in which year ? …………….

## PART 4

**Information about exposure to traumatic events.**

Did your child experience any of the items below during his/her life?
Please check ‘yes’ or ‘no’ for each item.

47. Has your child seen mutilated bodies and wounded people on television ___ no, ___ yes
48. Has your child heard or watched day raids of your family house ___ no, ___ yes
49. Has your child heard or watched night raids of your family house ___ no, ___ yes
50. Has your child heard or watched firing at your house by tanks and heavy artillery ___ no, ___ yes
51. Has your child heard or watched demolition of your family house ___ no, ___ yes
52. Has your child heard or watched shooting of a relative ___ no, ___ yes
53. Has your child heard or watched beating of a relative ___ no, ___ yes
54. Has your child heard the killing of a relative ___ no, ___ yes
55. Has your child watched the killing of a relative ___ no, ___ yes
56. Has your child heard or watched the arrest of a relative ___ no, ___ yes
57. Has your child heard or watched firing at neighbor’s house by tanks and heavy artillery
    ___ no, ___ yes
58. Has your child heard or watched shooting of a neighbor ___ no, ___ yes
59. Has your child heard the killing of a neighbor ___ no, ___ yes
60. Has your child watched the killing of a neighbor ___ no, ___ yes
61. Has your child heard or watched demolition of a friend’s house ___ no, ___ yes
62. Has your child heard or watched beating of a friend ___ no, ___ yes
63. Has your child heard or watched bombardment of other people’s houses by airplanes, helicopters or drones
    ___ no, ___ yes
