# Supplementary material for: Prevalence of functional constipation among Palestinian preschool children and the relation to stressful life events
Source: PLoS One. 2018 Dec 6;13(12):e0208571. doi: 10.1371/journal.pone.0208571 (PMC6283531; doi:10.1371/journal.pone.0208571)
Supplement: S2 Appendix — (PDF) [file pone.0208571.s002.pdf]

رقم الحالة..

7 mon-4 yrs

تقييم للإمساك والمغص بين أطفال اللاجئين الفلسطينيين

الذين تتراوح أعمارهم بين7 شهور الى 4 سنوات

التاريخ:____________ رقم ملف الطفل الصحي:________________

إسم العيادة الصحية:____

الاقليم:___الاردن ___الضفة الغربية ___غزة

الفئة العمرية:

0 7-12 mon
0 13-24 mon
0 25-36 mon
0 37-48 mon

الجزءالاول والثاني من الاستبيان يتم تعبئته بواسطة الباحث عن طريق مقابلة الأم بينما يستكمل الجزء الثالث والرابع من الاستبيان بواسطة الأم شخصيا

I. الجزء الاول

معلومات حول الطفل:

1. **الإسم:...**
2. **تاريخ الميلاد:...**
3. **الجنس: □ ذكر □ أنثى**
4. **كم عدد الأخوة والأخوات: عدد الأخوات ....عدد الأخوة ....**
5. **ما هو ترتيب الطفل في العائلة:..**
6. **الوزن الحالي:.. .... غرام**
7. **الطول الحالي : .... سم**

8. **أ) منحنى النمو (انظرمنحنى النمو للطفل): □ طبيعي □ يعاني من مشاكل في النمو**

   ب) اذا كان الجواب يعاني من مشاكل ,انظر لمنحنى النمو

   1. __ سمنة (>2SD) منحنى الوزن الطول
   2. __ ناقص وزن(<2SD) منحنى الوزن العمر
   3. __ تقزم(<2SD) منحنى الطول العمر
   4. __ نحول(<2SD) منحنى الوزن الطول

رقم الحالة...
7 mon-4 yrs

9. التاريخ الطبي العام: ....

10. هل يتطور نمو طفلك العقلي طبيعيا: ____لا____ نعم

II. الجزء الثاني

هذاالقسم يسأل عن حركةامعاءطفلك خلال الشهرين الماضيين. وهناك العديد من الكلمات لحركة الأمعاء،مثل" الخروج" ، "البراز" . عائلتك قد تستخدم كلمةأخرى خاصةعندما يتحدثون عن البراز.

11. خلال الشهرين الماضيين،عادة كم مرة طفلك يذهب للحمام للتبرز؟

1. ____ مرتان في الاسبوع أوأقل (اكمل للسؤال رقم 12)
2. ____ من 3 الى 6 مرات في الأسبوع
3. ____ مرة في اليوم
4. ____ من 2 الى 3 مرات في اليوم
5. ____ اكثر من 3 مرات في اليوم

(اكمل للسؤال رقم 13)

12. منذ متى طفلك لديه يتبرز بمعدل مرتين أو أقل في الاسبوع؟

1. ____ منذ أقل من شهرين
2. ____ منذ 2 6 أشهر
3. ____ منذ 6 اشهر الى سنة
4. ____ منذ 1 2 سنوات
5. ____ منذ 2 3سنوات
6. ____ منذ 3 4 سنوات

13. خلال الشهرين الماضيين، ما هو قوام براز طفلك؟ (اظهار الصور)

1. النوع الأول: قاسي جدا (الكتل الصلبة منفصلة)
2. النوع الثاني: صلب جاف
3. النوع الثالث: صلب عقدي
4. النوع الرابع: سلس وناعم
5. النوع الخامس: ناعم جدا ونقطي
6. النوع السادس: طري ومتصل
7. النوع السابع: مائي
8. يعتمد (برازه او برازها ليس متشابه دائما)

14. خلال الشهرين الماضيين، هل يعاني طفلك من الألم عند التبرز؟

1. ____ لا ابدا
2. ____ مرة في الشهرين
3. ____ من1 3 مرات في الشهر
4. ____ مرة في الاسبوع
5. ____ اكثر من مرة في الاسبوع

6.____ كل يوم

15.خلال الشهرين الماضيين، هل لدى طفلك اي تعسر(بذل مجهود)خلال حركة عملية التبرز؟

1.____ لا ابدا
2.____ مرة في الشهرين
3.____ من 1 3 مرات في الشهر
4.____ مرة في الاسبوع
5.____ اكثر من مرة في الاسبوع
6.____ كل يوم

16.في الشهرين الماضيين،هل كان لدى طفلك براز وكان كبيرجداًمما ادى الى انسدادالمرحاض؟(في الحجم والقطر)

1.____ لا
2.____نعم

17.بعض الأطفال لديهم عقدةالتبرزحتى عندما يتوفرالمرحاض. قديفعلون ذلك بشد أجسادهم أوعقدأرجلهم. خلال الشهرين الماضيين ,عندما يكون طفلك في المنزل،كم مرة عاني طفلك من عقدة التبرز؟

1.____ لا ابدا
2. ____ مرة في الشهرين
3.____ من مرة الى 3 مرات في الشهر
4.____ مرة في الاسبوع
5.____ أكثر من مرة في الاسبوع
6.____ كل يوم

18.أ) هل تم تدريب الطفل على استعمال المرحاض في المنزل وفي خارج المنزل؟

1.____ لا (اكمل الى السؤال21)
2.____ لا، فقط في البيت (اكمل الى السؤال21)
3.____ نعم

ب) اذا الجواب نعم في اي عمربالاشهر بدأ الطفل باستعمال المرحاض بدلا من الحفاظات؟ ....شهر

19.خلال الشهرين الماضيين للاطفال الذين اصبحوا نظيفين ويستعملون المرحاض،كم مرة عانى طفلك من اتساخ ملابسه الداخلية بالبراز؟

1.____ لا ابدا
2.____ مرة في الشهرين

3.___ من 1 3 مرات في الشهر
4.___ مرة في الاسبوع
5.___ اكثر من مرة في الاسبوع
6.___ كل يوم

20.خلال اليوم, للاطفال الذين اصبحوا نظيفين ويستعملون المرحاض، كم مرة عانى طفلك من اتساخ ملابسه الداخلية بالبول؟
1.___ لا ابدا
2.___ مرة في الشهرين
3.___ من مرة الى 3 مرات في الشهر
4.___ مرة في الاسبوع
5.___ اكثر من مرة في الاسبوع
6.___ كل يوم

21.خلال الشهرين الماضيين، كم مرة يخرج دم مع البراز؟
1.___ لا ابدا
2.___ مرة في الشهرين
3.___ من مرة الى 3 مرات في الشهر
4.___ مرة في الاسبوع
5.___ اكثر من مرة في الاسبوع
6.___ كل يوم

خلال الشهرين الماضيين ,هل عانى طفلك من الاعراض المذكورة ادناه ؟
22. الم في البطن ___لا___ نعم
23.تقيئ ___لا ___ نعم
24.فقدان شهية ___لا ___ نعم
25.فقدان وزن ___لا ___ نعم

26.هل برايك أن عادات التبرز عند طفلك يسبب له مشكلة؟
1.___لا
2.___نعم

27.هل تم فحص طفلك من قبل طبيب او ممرضة وتم إعلامك بأن طفلك لديه براز كثير داخله؟
1.___ لا
2.___ نعم

رقم الحالة..

7 mon-4 yrs

.28 أ)هل زرت في اي وقت مضى مركز الاونروا الصحي لان طفلك يعاني من مشاكل في البراز؟

1.___ نعم

2.___ لا، ذهبنا الى عيادة او مستشفى اخر

3.___ لا

ب) اذا كان الجواب نعم، في أي عمر بالاشهر؟...............................شهر

ج) اذا كان الجواب نعم، ما هي الفحوصات التي تمت من قبل الطبيب او الممرضة (والمزيد من الاجابات الممكنة)

1.___ فحص البطن

2.___ فحص فتحة الشرج من الداخل والخارج

3.___ فحوصات دم:..

4.___ اشعة:..

5.___ التراساوند:..

6.___ اخرى, حدد/ي :..

7.___ لا فحوصات

.29أ) في اي وقت مضى هل تمت معالجة طفلك بسبب الامساك من قبل طبيب او ممرضة الاونروا؟

1.___ نعم

2.___ لا

3.___ لا، تمت معالجته ذاتيا او نصيحة الصيدلية

ب) المعالجة تتضمن (المزيد من الاجابات الممكنة)

1.___ تقديم المشورة الغذائية

a. ___ أكل الياف اكثر

b. ___ أكل فواكه اكثر

c. ___ شرب ماء اكثر

d. ___ اخرى...

2.___ تقديم المشورة في سلوك الطفل

a. ___ تدريب على استعمال كبينة الاطفال

b. ___ العقاب

c. ___ المكافأة

d. ___ اخرى

3.___ المسهلات عن طريق الفم

a. ___ حبوب نوع..

b. ___ شراب نوع..

c. ____ بودرة نوع...

4.____ مسهلات عن طريق الشرج
a. ____ غلسرين تحاميل
b. ____ تحاميل أخرى
c. ____ حقنة شرجية

5.____اعشاب طبية ,حدد\ي ....
6.____ أخرى, حدد\ي..

30.خلال السنة الماضية، هل تعرضت الى اي عنف اسري (جسدي أولفظي) في المنزل؟
1. ____ لا أبدا
2. ____ مرة واحدة في السنة
3. ____ اكثر من مرة في السنة
4. ____ مرة واحدة في الشهر
5. ____ أكثر من مرة في الشهر
6. ____ مرة في الاسبوع
7. ____ اكثر من مرة في الاسبوع
8. ____ كل يوم

31 خلال السنة الماضية، هل تعرض طفلك الى اي عنف اسري(جسدي أو لفظي) في المنزل؟
1. ____ لا أبدا
2. ____ مرة واحدة في السنة
3. ____ اكثر من مرة في السنة
4. ____ مرة واحدة في الشهر
5. ____ أكثر من مرة في الشهر
6. ____ مرة في الاسبوع
7. ____ اكثر من مرة في الاسبوع
8. ____ كل يوم

رقم الحالة..
7 mon-4 yrs

الفئة العمرية:

0 7-12 mon
0 13-24 mon
0 25-36 mon
0 37-48 mon

III. الجزء الثالث: معلومات عن العائلة

32. عمر الأم...سنة
33. عمر الأم عند الزواج...سنة
34. عدد سنوات دراسةالأم..سنة

35. عمل الأم:
1. لا يعمل
2. عامل غير ماهر
3. عامل ماهر
4. موظف مؤسسة أو دائرة حكومية
5. تاجر
6. حامل شهادة يعمل بها
7. وظيفة أخرى..

36. عمر الأب ....سنة
37. عمر الاب عند الزواج..سنة
38. عدد سنوات دراسة الاب..سنة

39. عمل الأب:
1. لا يعمل
2. عامل غير ماهر
3. عامل ماهر
4. موظف مؤسسة أو دائرة حكومية
5. تاجر
6. حامل شهادة يعمل بها
7. وظيفة أخرى..

40. دخل العائلة:
الاردن:
1. اقل من 300 JD
2. من 300 500 JD
3. اكثر من 500 JD

رقم الحالة..
7 mon-4 yrs

الضفة الغربية, غزة:
1.اقل من 1500 شيكل
2. 1500 2500 شيكل
3. اكثر من 2500 شيكل

41. هل تشعر ان دخلك كافي لحاجاتك الاساسية وحاجات عائلتك؟
1. لا ____
2. نعم ____

42. هل تتحمل عائلتك أي ديون مالية؟
1. لا ____
2. نعم ____

43. كيف علاقتك مع زوجك؟
1. جيد جدا
2. جيد
3. سيء
4. سيء جدا

44. كيف علاقتك مع افراد الاسرة الاخرين (أمك ,أبوك,حماك,حماتك, أخوة)اللذين يعيشون معكم في البيت؟
1. جيد جدا
2. جيد
3. سيء
4. سيء جدا
5. لا يسكن معنا أحد من أفراد الاسرة

45. مكان السكن:
1. مخيم لاجئين
2. المدينة
3. منطقة ريفية او قرية

46.أ) هل كانت عائلتك مجبرة على تغيير مكان إقامتها بسبب الوضع الأمني أو لأسباب أخرى؟
1. لا ____
2. نعم____

ب) اذا كان الجواب نعم____ حددا\ ي أي سنة_____

رقم الحالة..

7 mon-4 yrs

IV. الجزءالرابع: معلومات حول التعرض لأحداث مؤلمة

هل تعرض طفلك لأي من الأحداث التالية خلال أي وقت في حياته؟

يرجى الإجابة بنعم او لا لكل سؤال

47. شاهد طفلك جثث مشوهة ومجروحة على التلفزيون ____لا____نعم
48. شاهد طفلك غارات نهارية على منزلك ____لا ____نعم
49. شاهد طفلك غارات ليلية على منزلك ____لا ____نعم
50. شاهد طفلك اوسمع اطلاق نار بالدبابات والمدفعية الثقيلة على منزلك ____لا____نعم
51. شاهد طفلك او سمع هدم منزلك ____لا ____نعم
52. شاهد طفلك او سمع اطلاق نارعلى احد أقربائك ____لا ____نعم
53. شاهد طفلك او سمع ضرب احد اقربائك ____لا ____نعم
54. سمع طفلك قتل احد أقربائك ____لا ____نعم
55. شاهد طفلك قتل احد اقربائك ____لا ____نعم
56. شاهد طفلك او سمع اعتقال احد أقربائك ____لا ____نعم
57. شاهد طفلك او سمع اطلاق ناربالدبابات والمدفعية الثقيلة على منزل جيرانك ____لا ____نعم
58. شاهد طفلك أو سمع اطلاق نار على احد من جيرانك ____لا ____نعم
59. سمع طفلك قتل احد جيرانك ____لا ____نعم
60. شاهد طفلك قتل احد جيرانك ____لا ____نعم
61. شاهد طفلك او سمع هدم بيت احد أصدقائك ____لا ____ نعم
62. شاهد طفلك او سمع ضرب احد أصدقائك ____لا ____نعم
63. شاهد طفلك او سمع قصف منازل بطائرات عادية او طائرات هليوكبتر أو طائرة بدون طيار ____لا____نعمٍ
